# Soy and Frequent Dairy Consumption with Subsequent Equol Production Reveals Decreased Gut Health in a Cohort of Healthy Puerto Rican Women

**DOI:** 10.3390/ijerph18168254

**Published:** 2021-08-04

**Authors:** Mercedes Y. Lacourt-Ventura, Brayan Vilanova-Cuevas, Delmarie Rivera-Rodríguez, Raysa Rosario-Acevedo, Christine Miranda, Gerónimo Maldonado-Martínez, Johanna Maysonet, Darlene Vargas, Yelitza Ruiz, Robert Hunter-Mellado, Luis A. Cubano, Suranganie Dharmawardhane, Johanna W. Lampe, Abel Baerga-Ortiz, Filipa Godoy-Vitorino, Michelle M. Martínez-Montemayor

**Affiliations:** 1 Department of Biochemistry, School of Medicine, Universidad Central del Caribe, Bayamón 00956, Puerto Rico; mercedes.lacourt@uccaribe.edu (M.Y.L.-V.); raysa21@gmail.com (R.R.-A.); darlene.vargas@upr.edu (D.V.); lcubano@gmail.com (L.A.C.); 2 Department of Microbiology and Medical Zoology, Medical Sciences Campus, University of Puerto Rico, San Juan 00921, Puerto Rico; vcbycedin@gmail.com (B.V.-C.); filipa.godoy@upr.edu (F.G.-V.); 3 Department of Biology, University of Puerto Rico, Bayamón 00959, Puerto Rico; delmarie.rivera@upr.edu; 4 Retrovirus Research Center, Internal Medicine Department, School of Medicine, Universidad Central del Caribe, Bayamón 00956, Puerto Rico; christine.miranda@uccaribe.edu (C.M.); geronimo.maldonado@gmail.com (G.M.-M.); jmaysonet@cccupr.org (J.M.); fabiyelly@yahoo.com (Y.R.); huntermellado@gmail.com (R.H.-M.); 5 Hematology and Oncology Group, HIMA-San Pablo Bayamón Hospital, Bayamón 00961, Puerto Rico; 6 Department of Biochemistry, Medical Sciences Campus, University of Puerto Rico, San Juan 00921, Puerto Rico; su.d@upr.edu (S.D.); abel.baerga@upr.edu (A.B.-O.); 7 Fred Hutchinson Cancer Research Center, Division of Public Health Sciences, Seattle, WA 98109, USA; jlampe@fredhutch.org

**Keywords:** equol, soy, dairy, gut microbiota, Puerto Rican women

## Abstract

The U.S. Hispanic female population has one of the highest breast cancer (BC) incidence and mortality rates, while BC is the leading cause of cancer death in Puerto Rican women. Certain foods may predispose to carcinogenesis. Our previous studies indicate that consuming combined soy isoflavones (genistein, daidzein, and glycitein) promotes tumor metastasis possibly through increased protein synthesis activated by equol, a secondary dietary metabolite. Equol is a bacterial metabolite produced in about 20–60% of the population that harbor and exhibit specific gut microbiota capable of producing it from daidzein. The aim of the current study was to investigate the prevalence of equol production in Puerto Rican women and identify the equol producing microbiota in this understudied population. Herein, we conducted a cross-sectional characterization of equol production in a clinically based sample of eighty healthy 25–50 year old Puerto Rican women. Urine samples were collected and evaluated by GCMS for the presence of soy isoflavones and metabolites to determine the ratio of equol producers to equol non-producers. Furthermore, fecal samples were collected for gut microbiota characterization on a subset of women using next generation sequencing (NGS). We report that 25% of the participants were classified as equol producers. Importantly, the gut microbiota from equol non-producers demonstrated a higher diversity. Our results suggest that healthy women with soy and high dairy consumption with subsequent equol production may result in gut dysbiosis by having reduced quantities (diversity) of healthy bacterial biomarkers, which might be associated to increased diseased outcomes (e.g., cancer, and other diseases).

## 1. Introduction

For the past decade, there has been a trend to use functional foods to improve health. The term functional food is used to describe a food or food ingredient that contains non-nutrient bioactive compounds that may promote health benefits to individuals [1]. The CDC (Centers for Disease Control and Prevention) and WHO (World Health Organization) have recommended and published a series of nutritional guidelines to promote the consumption of foods that are good for the wellbeing of individuals (i.e., have preventive or protective benefits) and foods that might be harmful, thus might have increased carcinogenic potential [2,3]. Importantly, these nutrition guidelines do not consider ethnicity or gut microbiota diversity, which are additional factors that could also play a role in increased disease propensity. Humans have evolved with a microbiome from birth, and with differential gut colonization due to diet and lifestyle. This results in different processes that may cause dysbiosis (i.e., altered microbiota) of the microbial communities [4,5]. In recent years, studies have evaluated the role of the microbiota as an ecosystem [4]. Specifically, the gut microbiota in humans harbor species that assist the digestive process by fermenting and metabolizing dietary components and signaling molecules, and these may affect the host positively or negatively. The gut holds ~10^12^ bacterial cells per gram of colonic matter, and it is composed of ~400 different species [6,7]. The gut microbiota contributes to ~3.3 million genes, which is ~150 times the size of the human genome. This collection of genes is called the microbiome, which can affect all aspects of the host including the immune system, metabolome, physiology, and behavior. Certain factors can alter the stability of the individual’s microbiota such as antibiotics, age, diet, and genetics [8]. The field of microbial ecology studies the composition and structure of microbial communities by high-throughput sequencing of 16S rRNA variable region gene fragments (abbreviated 16S) or through metagenomics, which sequences all given genomic DNA from a sample. Herein, we focus on characterizing the gut microbiota via 16S of healthy Puerto Rican women, in the context of equol, an isoflavone-derived microbial metabolite produced by gut bacteria in some individuals. Our previous research suggests equol increases breast cancer progression [9,10].

For decades, the role of phytochemicals (e.g., isoflavones, phytoestrogens) in health has been controversial [11,12,13,14,15,16,17,18]. Isoflavones are phytochemicals found in legumes, particularly in soybeans, with roles such as UV protection, anti-microbial, and induction of rhizobial nodules [19]. Isoflavones are structurally similar to estrogens and for this reason, have been extensively studied as a possible therapy for a myriad of medical conditions, but also as a plausible causative of endocrine disruption and cancer. Some of the most common phytoestrogens are daidzein, genistein, and glycitein [7]. Daidzein has been used in studies of stroke recovery, as a possible protection agent against cisplatin-induced nephrotoxicity, and as a tumor growth inducer in mouse models [9,20,21]. Importantly, these isoflavones undergo transformation into several secondary metabolites (e.g., equol) by specific gut bacteria found in some mammalian species [22,23].

Equol was isolated for the first time in 1932 from pregnant mare urine and it takes its name from the sample source [24]. Equol was first isolated from human urine in 1982 [25], and has been identified in several animal species like rodents, canines, and non-human primates [26,27]. Not every animal can convert daidzin/daidzein to equol to the same degree of efficiency [28], and its synthesis from daidzein might have dihydrodaidzein (DHD) as an intermediate metabolite [29]. Isoflavones and its bacterial metabolites are mostly excreted, but some may be found in animal products like milk and dairy [30,31,32]. Equol has two enantiomers: R-equol and S-equol; in humans, the gut microbiota exclusively synthesizes S-equol from daidzein [33]. Studies show that 20–60% of the population have the bacteria essential for transforming daidzein to equol, where the prevalence of equol producers is 20–30% in Western countries and 40–60% in Asian countries [34,35,36,37]. The inter-individual differences and structure of the gut microbiota regulates the ability to produce equol and other isoflavone metabolites [35], and studies show inconsistencies about the health benefits of equol, probably caused by the differences in the prevalence of equol producers between the Western and Asian countries [18]. Some of the possible benefits that have been studied are anti-inflammatory, ease of menopause symptoms such as hot flashes and osteoporosis, lower cardiovascular disease risk, immune system, and as antioxidants [13,18,38,39]. However, several studies including our own have explored the potential role of equol, daidzein, and other isoflavones such as cell proliferation, tumor progression, and proto-oncogene induction promoter in in vivo and in vitro models [9,10,14,40,41]. Another bacterial metabolite from daidzein is O-desmethylangolensin (ODMA), which was isolated in the 1980s from human urine, but it was not until the 1990s that it was identified as a bacterial product [30,42,43]. Studies have shown that most of the population (80–90%) has the gut bacteria to produce it [28,37]. ODMA’s anticancer activity has been studied in vitro in breast and hepatic cells [44], while its relationship with obesity shows that those that are ODMA non-producers are more likely to be obese than ODMA producers [45,46].

The Puerto Rican diet is highly based on legumes, especially beans, and includes frequent consumption of dairy products, a possible source of equol. Therefore, this study aims to investigate if healthy premenopausal Puerto Rican women possess equol producing gut microbiota, and whether they are equol producers, based on their regular dietary habits. To our knowledge, this is the first study to explore equol and other plant metabolites in Puerto Ricans and characterizes the gut microbiota that generates these metabolites. Our study showed that equol producers (25%) have lower amounts of “healthy” bacterial biomarkers. This study will serve as a starting point for future research to elucidate the role of equol on the health of Puerto Ricans, especially in breast cancer incidence.

## 2. Materials and Methods

### 2.1. Study Subjects

**Figure 1:**
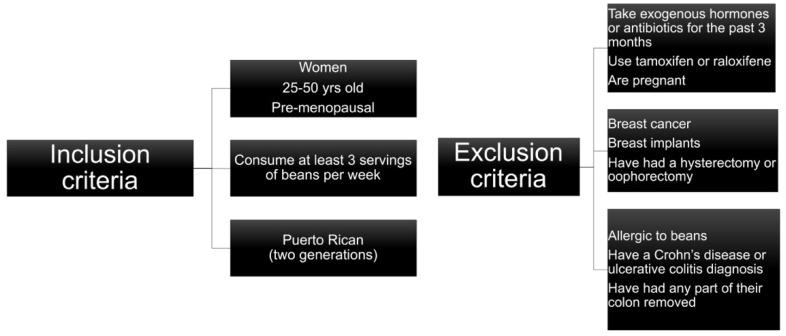
Inclusion and exclusion criteria of the subjects recruited at the three sites (HURRA, PRHOG, PRCTRC).

A total of 93 adult female participants that visited the Internal Medicine Clinic at the Ramon Ruiz Arnau University Hospital (HURRA), Bayamón, Puerto Rico; the Puerto Rico Hematology and Oncology Group (PRHOG) at the HIMA San Pablo Hospital, Bayamón, Puerto Rico; and the Puerto Rico Clinical and Translational Research Consortium (PRCTRC) clinic at the University of Puerto Rico Medical Sciences Campus, San Juan, Puerto Rico were recruited from August 2010 to June 2011. These women attended these clinics for a regular medical checkup, where the health professional staff identified the possible study candidates. Of these, we excluded 13 subjects whose stool samples were not collected. Therefore, 80 subjects were included in the final analysis (Figure 1).

### 2.2. Lifestyle Questionnaire

Once consent was obtained for the subject, the clinical coordinator administered an initial survey with 11 questions that determined subject eligibility to participate in the study. Eligible subjects then answered a lifestyle questionnaire that consisted of 51 questions to assess demographic and anthropometric information, nutritional regimen (e.g., type of food and frequency of consumption), and physical activity.

### 2.3. Urine Concentration of Metabolites

Upon obtaining consent, and after the initial survey and lifestyle questionnaire assessment, a spot urine sample was collected and stored at −20 °C until ready to analyze. Urine samples (*N* = 80) were analyzed for genistein, daidzein, equol, O-desmethylangolensin (ODMA), and dihydrodaidzein (DHD), and the enterolignans, enterodiol, and enterolactone. Deuterated analogues of each compound were added to the urine samples (4 mL) prior to extraction. Samples were enzymatically hydrolyzed with beta-glucuronidase (Sigma), extracted with ether, evaporated to dryness, derivatized with 15% BSTFA with 1% TMCS (Thermo Scientific), and analyzed by GCMS in the selected ion monitoring mode using a 6890N Network GC System with 7683 Series auto-injector and 5975 Inert XL Mass Selective detector (Agilent Technologies) [29,47]. The instrument configuration included a fused silica capillary column (12 m × 0.20 mm × 0.33 µm) poly(dimethyl siloxane) bonded phase SPB-1 (Supelco). Helium was used as the carrier gas (flow rate 1.2 mL/min). The oven temperature was held at 100 °C for 1 min, heated 20 °C/min to 290 °C, and held for 5.5 min. The injector, ion source, and interface temperatures were 250 °C, 200 °C, and 290 °C, respectively. Method detection limits were 0.003125 ng/μL for all compounds with 4 mL urine. The interassay coefficients of variation for all metabolites were <6%. Equol producers were classified as those having detectable equol concentrations (≥0.003125 ng/μL urine).

### 2.4. Gut Microbiota Data Production and Analysis by Next Generation Sequencing (NGS)

#### 2.4.1. Genomic DNA Extractions from Fecal Samples

Fecal samples were collected upon obtaining consent, and after the initial survey and lifestyle questionnaire assessment. If the subject could not provide the sample on site, they were given a stool sample collection kit with instructions to collect, store, and return the fecal sample. The samples needed to be collected within the next 24 h of recruitment, stored at 4 °C, and returned immediately to the recruitment facility. Once in the facility, samples were stored at −20 °C until ready to analyze. Total genomic DNA (gDNA) was extracted using the QIAamp DNA Stool Kit (Qiagen Inc, CA, USA) by following the manufacturer’s instructions. DNA concentration was quantified using a Nanodrop 1000 (Thermo Scientific, MA, USA). A total of 20 samples were selected based on the urine GCMS data [10 from non-producers (randomly chosen from the subject samples that resulted in values below detection limit, BDL for equol) and 10 equol producers (had detectable equol values, 0.007–0.747ng/μL urine)]. Three of the ten equol producers’ samples did not comply with the stringent QC tests; therefore the remaining seven equol-producer samples were used for the final analysis (*n* = 17).

#### 2.4.2. 16S rDNA Amplifications and Illumina Sequencing

DNA extracted from fecal samples were normalized to 4nM during l6S library prep. We amplified the V4 hypervariable region of the 16S ribosomal RNA marker gene (~291 bp) using the universal bacterial primers: 515F (5′ GTGCCAGCMGCCGCGGTAA 3′) and 806R (5′ GGACTACHVGGGTWTCTAAT 3′) in the Earth Microbiome Project (http://www.earthmicrobiome.org/emp-standard-protocols/16s/ accessed on 1 January 2020) [48] using previously reported conditions [49]. We used the Illumina MiSeq Reagent Kit 2 × 250 bp to sequence the 16S amplicons. The 16S rDNA reads were deposited in QIITA [50] Bioproject ID 12663 and available at the European Nucleotide Archive EBI Study: ERP129008.

#### 2.4.3. Read QC and Bioinformatic Analyses

The 16S rRNA Raw FASTQ sequence files were deposited and processed IN QIITA [50] using per-sample FASTQs with a Phred offset of 33, min_per_read_length_fraction of 0.75 and default parameters for error detection using Split libraries FASTQ. Sequences were trimmed to 250 bp and reference operational taxonomic units (OTUs) were defined with a closed reference approach using the SILVA reference database [51] with a minimum similarity threshold of 97% and corresponding taxonomy assignment using the default parameters in QIITA. Singletons (OTUs with less than three reads), sequences matching chloroplasts, mitochondria, and unassigned sequences were removed from downstream analyses using QIIME2 [52].

Beta diversity analyses of microbial communities were done by computing the pairwise Bray–Curtis distances between samples and plotted using non-metric multidimensional scaling (NMDS). Alpha diversity and taxonomic plots: Taxonomic barplots, alpha richness Chao1 (estimated number of OTUs), and diversity boxplots (Shannon index of equitability [53]) were built using R’s ggplot2 package [54].

The rarefaction level used in the 16S rDNA analyses for the equol 40% core (OTUs present in at least 40% of samples) was 23,200 reads for soy and equol joint analyses for which the 40% core level were 4236 reads. Metadata variables used in the analyses included equol producers and non-producers; those who consumed beans and soy and are equol producers and non-producers. Boxplots of specific taxonomic OTU changes among genus were plotted libraries in the ggplot2 package [54] in R (Team, 2008) https://www.r-project.org/ (accessed on 1 January 2021).

Additionally, we used linear discriminant analysis (LDA) with the LefSe algorithm [55] to detect biomarkers between the metadata categories by using a non-parametric factorial Kruskal–Wallis (KW) sum-rank test, Wilcoxon rank-sum test, and LDA.

Network pathway analyses were done using QIIME2’s PICRUST2 function. Using the PICRUST2 output metabolic pathway table, we filtered out all pathways that had less than a 0.05 raw p-value significance scores and used QIIME1 to transform the pathway inferred reads into nodes and edges and developed the network using Cytoscape [56].

### 2.5. Statistical Analysis

Differences between producers and nonproducers of the daidzein metabolites in demographic, anthropometric, and lifestyle factors were assessed with the use of independent samples t tests, chi-square analyses, and Fisher’s exact tests. Diagnostic for normality criteria was performed using the Shapiro–Francia estimator. Presence of outliers were verified via Dixon’s test. Data distribution was confirmed using central tendency and dispersion measures. Inter-dependence statistical significance was verified using a two-stage correlation matrices approach. Zero order correlations were calculated to monitor variable association with no control. Partial correlations were calculated to observe the coefficient change in the presence of a control variable. The significant level (α) was set to ≤0.05). R v.3.6 (Team R: A language and environment for statistical computing) was used.

Beta diversity was assessed for statistical significance between sample groups using the PERMANOVA test [57]. The *p*-value in a PERMANOVA test was determined through permutations, and the test statistic was calculated directly from the distance matrix. For alpha diversity, we used the script compare_alpha_diversity.py in QIIME to compare the diversity between groups of samples in a given metadata category by performing a *t*-test using non-parametric, Monte Carlo permutations, and the results were corrected for multiple comparisons using the Bonferroni post-hoc test.

### 2.6. Ethics Statement

This study was performed in accordance with the ethical standards of the Declaration of Helsinki, and approved by the Institutional Review Board committee at Universidad Central del Caribe School of Medicine (2010-024) for HURRA and PRHOG sites, and at the University of Puerto Rico Medical Science Campus (A9560112) for the PRCTRC site. All subjects provided written informed consent for this study.

## 3. Results

### 3.1. Demographic, Anthropometric, Lifestyle and Dietary Factors

Of the 93 women who attended the clinic visits, our recruited cohort who provided urine and fecal samples had a sample size of 80 women. Of these women, all 80 completed the health and demographics questionnaire, and 79 completed the anthropomorphic information. Subjects’ mean age at recruitment was 38.9 (SD 7.8) years old, while the mean BMI was 29.7 (SD 8.09), which is in the range of overweight and obese. The mean age of first menarche was 12.3 (SD 1.89) years old, and most of the women had a university degree, did not smoke, had more frequent constipation, and no family history of breast cancer. Moreover, most women reported having an income between $20,000–$30,000 per year and having private health insurance (Table 1).

The percentage of consumption or non-consumption of major food components in the Puerto Rican standard diet of the 80 participants is summarized in Table 2. Most of the study subjects consumed meat (beef, pork, poultry, and or lamb), fish, beans, fruits, vegetables, and dairy. Regarding dairy consumption frequency, 50% of the subjects consumed dairy four times or more per week, 46.3% 1–3 times per week, and 2.5% had never ingested it. As expected, most of the subjects ate beans (86%), which were placed under a different category from soy. While soy consumption (58.8%) was lower than bean consumption, the numbers were not that different from consumers (41%).

### 3.2. Urinary Metabolites

Among the 80 women, twenty (25%) of the participants were equol producers (levels ranged from 0.007–0.747 ng/μL urine), fifty-four (68%) produced ODMA, eighteen (23%) produced DHD, while seventy-three (91%) and seventy-six (95%) subjects produced genistein and daidzein, respectively. GCMS analysis was also performed to analyze non-flavonoid enterolignan production where sixty (75%) women produced enterodiol, while 77 of the 80 women (96%) produced enterolactone.

To assess the relationship between the urine metabolites, a Pearson correlation coefficient (r) was computed. Results demonstrated (Table 3) that the urine metabolites displayed a positive correlation. Weaker metabolite correlations were with ODMA vs. enterodiol, daidzein vs. DHD. Metabolites with high correlations were ODMA vs. daidzein, ODMA vs. genistein, daidzein vs. genistein, and equol vs. genistein. In addition, both enterolignans showed a significant correlation between themselves.

### 3.3. Microbial Composition Analysis and Metabolic Pathway Inference Results from Fecal Samples of Healthy Puerto Rican Women Correlated with Equol Production and Soy Consumption

**Figure 2:**
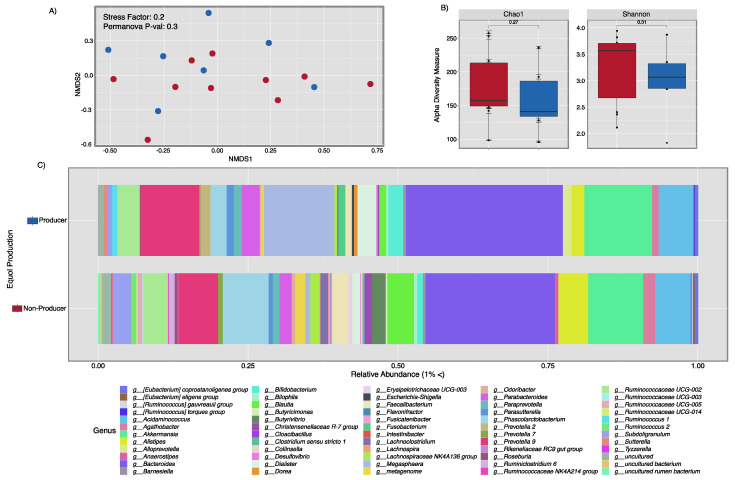
Microbiota diversity between equol producers and equol nonproducers. Fecal samples from 17 women, (*n* = 7 equol producers and *n* = 10 equol non-producers) underwent 16S rRNA sequencing and were analyzed for microbial diversity. No significant differences in beta-diversity were found (Panel **A**). Although alpha-diversity showed non-significant differences (Panel **B**), the gut microbiota from equol non-producers has a slightly higher diversity. Taxonomy bar plots between non-producers and equol producers showed a similar composition with slightly higher dominance of *Akkermansia* among the equol producers, and a higher dominance in *Bacteroides* in the equol non-producers (Panel **C**).

To characterize the gut microbiota in equol producers (*n* = 10) vs. equol non-producers (*n* = 10), we extracted gDNA from fecal samples collected during recruitment. gDNA extracted from three of the ten equol producer samples did not comply with the Q/C analysis, thus only seven samples were used for the remaining analyses (*n* = 17). Beta-diversity was not significantly different when comparing equol vs. equol non-producers (*p =* 0.34) (Figure 2A). We found a slightly higher gut diversity in equol non-producers, although not significantly different (Chao1 *p =* 0.27; Shannon *p =* 0.31). These data suggest that equol production in healthy subjects may contribute to reducing gut diversity (Figure 2B). In terms of bacterial composition, equol producers had lower amounts of bacterial biomarkers known to be beneficial taxa such as *Bacteroides* spp., *Faecallibacterium* spp., and few *Butyrivibrium spp.* reads. However, we found higher abundances of *Acidominococcus* spp., *Phascolarctobacterium* spp., and *Alistipes* spp. in non-producers while in equol producers, there was a higher dominance of *Akkermansia* spp., *Prevotella 9*, and *Megasphera elsdenii* (Figure 2C).

**Figure 3:**
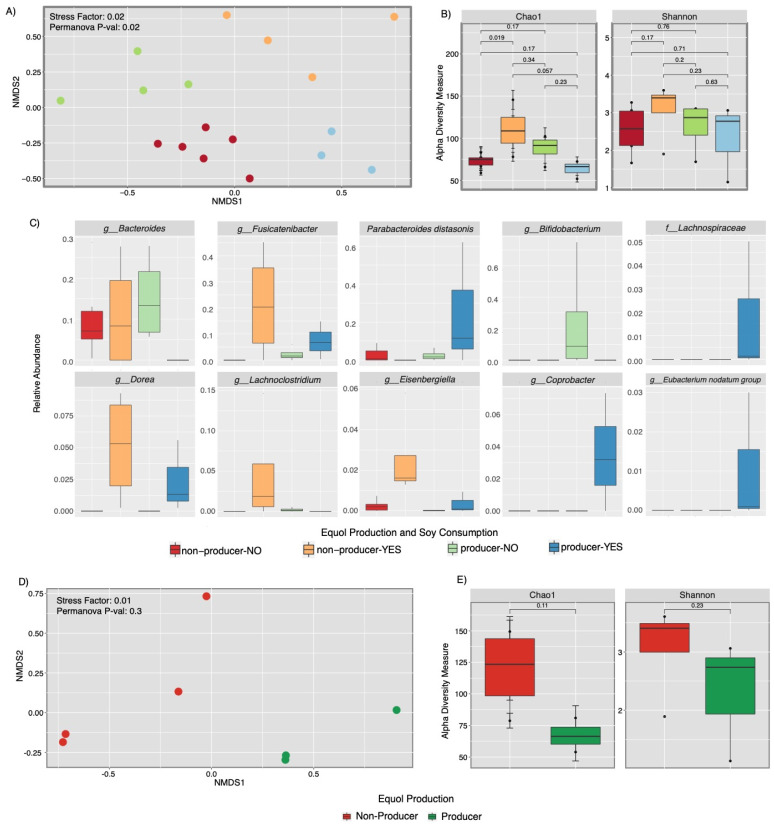
Fecal microbial community patterns according to soy consumption and equol production. Panels (**A**–**C**) represent all participants stratified by soy consumption and equol production (*n* = 17 samples). Panel C represents taxa that changed significantly in each category (*p*-value < 0.05. Panels (**D**,**E**) (*n* = 7) represent only participants that consumed soy and produced equol or not. Fecal bacterial community diversity appeared to be significantly higher among those who did not produce equol).

Fecal microbial communities displayed significant differences among equol-producers and equol non-producers once they were stratified by soy consumption (Figure 3). Our results showed significant structural differences (beta diversity) of the microbial communities and clear separation of subjects that did not consume soy to the negative axis (left), while soy consumption samples to the positive (right) axis (*p* < 0.02) (Figure 3A). Moreover, our results showed differences in diversity among equol non-producers (Chao 1 *p* < 0.019) (Figure 3B). Equol producers that did not consume soy had a higher dominance in *Bacteroides* spp. and *Bifidobacterium* spp. (Figure 3C). Equol non-producers that did consume soy had a greater abundance of *Dorea* spp., *Fusicatenibacter* spp., *Eisenbergiella* spp., and *Lachnoclostridium* spp. (Figure 3C). When samples from soy consumer participants (*n* = 7) were stratified by equol production, we found a significant sample dispersion with the separation of equol non-producers to the negative axis, while equol producers shifted to the positive axis although these were not significant (*p =* 0.3) (Figure 3D). We did not find significant differences between alpha richness (Chao1 *p* = 0.11) or diversity (Shannon *p* = 0.23) (Figure 3E), despite the increase in diversity among equol non-producers.

In the interest of understanding whether bean consumption was associated with microbiota diversity and equol production, we sought to select participants who consumed beans, produced equol, and their associated gut microbiota changes. Our studies revealed that thirteen of the twenty samples chosen for gut microbiota analysis from healthy women consumed beans. Furthermore, eight (62%) of these women were equol non-producers, while five (38%), were equol producers. Our results showed that bean consumers that did not produce equol had a distinct community structure (as driven by composition) than equol producers (*p* = 0.07) (Supplementary Figure S1A). Furthermore, our data showed no significant differences in alpha diversity (Supplementary Figure S1A). Additionally, we also investigated the microbiota profiles among all 17 participants according to their bean and soy consumption (both, either, or none), and equol production (Supplementary Figure S2). Our analysis revealed no significant differences in alpha-diversity nor beta diversity, despite some differences in composition (Supplementary Figure S2A). We found higher dominance of *Akkermansia* among the equol producers who consumed both soy and beans (Supplementary Figure S2C).

**Figure 4:**
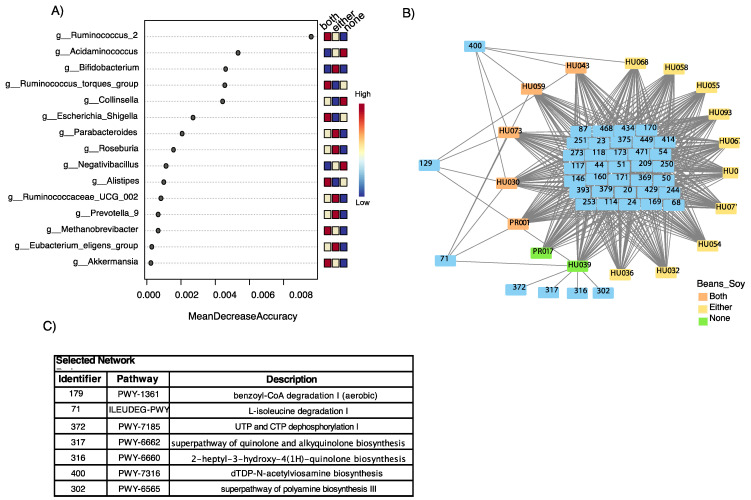
Linear discriminant analysis effect size (LEFsE) of bacterial communities. LEfSe plot showing possible bacterial markers of consumption of both beans and soy Panel (**A**). Pathway network identifies similarities among soy and/or bean consumers in terms of the potential biochemical pathways of the microbiota. Panel (**C**). Table associating bacterial taxa to the pathways identified in the network in Panel (**B**).

To test which bacterial communities were predominantly found among bean and soy consumers, we performed linear discriminant analysis effect size (LEFsE). LEFsE determines the features (e.g., operational taxonomic units) most likely to explain differences between classes by coupling tests for statistical significance with additional tests encoding biological consistency and effect relevance [55]. Our results show that healthy individuals who consumed beans and soy mostly had *Ruminococcus, Shiggela, Alistipes*, and *Akkermansia* in their gut microbiota (Figure 4). Our metabolic inference network analysis presented many shared pathways among all samples including specific pathways correlated to subjects that both did and did not consume beans or soy. In fact, *Ackermansia, Ruminococcus,* and *Methanobacter* participated in L-isoleucine degradation and benzoyl-CoA degradation among the bean and soy consumers.

**Figure 5:**
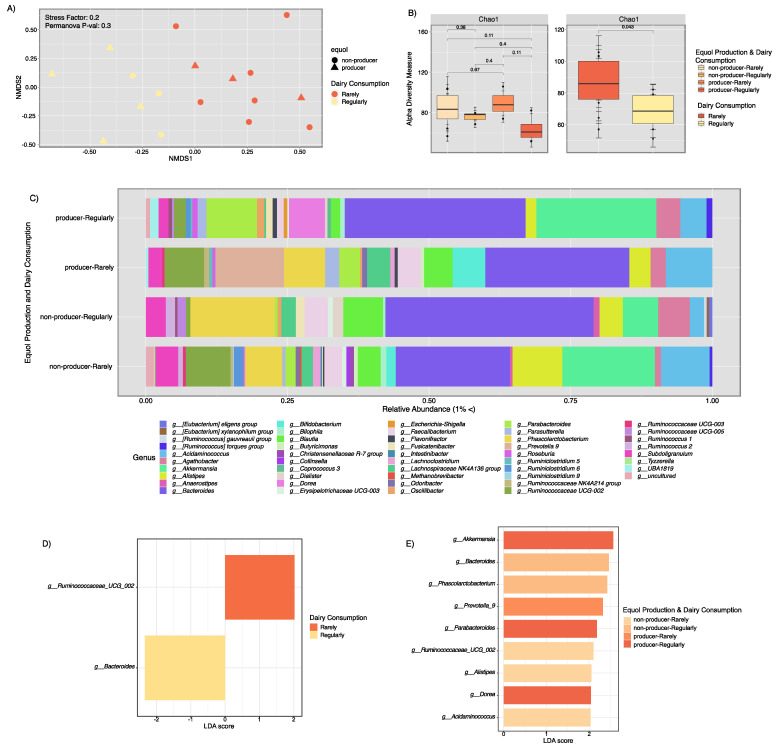
Fecal microbial community patterns according to dairy consumption frequency. Panel (**A**) depicts beta-diversity patterns while panel (**B**) shows alpha diversity (estimated Chao1 richness). Panel (**C**) depicts community composition for dairy consumption and equol production combined. Panels (**D**,**E**) show bacterial taxa that were significantly dominant according to dairy consumption and/or equol production using LeFSE.

Finally, to test the microbiota associated with equol production and dairy consumption, we included the data collected on the consumption and frequency of dairy consumption. Beta diversity analysis showed structural differences between the samples of women who consumed dairy rarely versus regularly, which were divided by the NMDS1 axis, although these were not significant [Stress factor 0.21 and Permanova *p =* 0.5 (dairy and equol cat) *p =* 0.3 (dairy consumption)]. Alpha richness analysis using the Chao1 metric for both dairy consumption and concatenated equol production presented no significant differences, (Figure 5B), however, when analyzing dairy consumption alone, we found that women who rarely consumed dairy displayed a richer microbiota than women who consumed dairy regularly *p* < 0.04).

Taxonomical assessment performed by dividing equol producers and dairy consumption found that women who regularly consumed dairy had less *Bacteroides*. Furthermore, these women presented a higher abundance of *Lachnoclostridium* and *Enterobacteriacea.* Equol producers that rarely consumed dairy presented a higher abundance of *Prevotella, Lachnospiraceae* NK4A136, *Dialister, Odoribacter,* and *Acidaminococcus*, from which *Dialister, Odoribacter*, and *Oscillibacter* are known equol producing bacteria [58]. Equol non-producers who regularly consumed dairy had a higher abundance of *Bacteroides, Blautia, Phascolarctobacterium*, and *Agathobacter* genera (Figure 5C). Linear discriminant analysis was used to investigate health biomarkers based on the taxonomical groups that held higher abundance. Our results showed that women who consumed dairy regularly had a higher abundance of *Bacteroides* and the ones who consumed dairy rarely presented a higher abundance of *Ruminococcaceae* UCG_002 (Figure 5D). Moreover, equol non-producers who rarely consumed dairy had a higher abundance of *Acidaminococcus*, *Alistipes*, and *Ruminococcaceae* UCG_002, while equol non-producers who regularly consumed dairy displayed a higher abundance of *Bacteroides* and *Phascolarctobacterium*. Equol producers who rarely consumed dairy presented a higher abundance of *Prevotella*, while equol producers who consumed dairy regularly had a higher abundance of *Akkermansia*, *Parabacteroides*, and *Dorea*.

## 4. Discussion

For the past decades, the consumption of isoflavones, particularly from soy, has increased, especially under the perception that it is good for one’s health when comparing the low rates of cancer in Asian versus Western populations. It is important to investigate whether Western populations might benefit from isoflavone consumption like their Eastern counterparts or in contrast, whether people presenting with particular phenotypes should abstain from soy consumption. Our previous findings using in vitro and in vivo cancer models suggest that the intake of combined soy isoflavones (genistein, daidzein, and glycitein) can promote cancer cell proliferation, tumor progression, metastasis, and upregulation of oncogenes [9]. Furthermore, we also reported that the metabolite equol is one of the main contributors of increased BC progression by displaying metastasis in mice and invasion processes in cancer cell models [10]. With the current study, we aimed to document, for the first time, the prevalence of equol production in a group of healthy Puerto Rican women and characterize the gut microbiota present within this population, given their increased risk of developing cancer.

Contrary to previous studies [34], in the current study, we did not perform a soy challenge in our subjects, because we aimed to assess equol production only by the usual diet consumed by healthy participants. The Puerto Rican diet is based heavily on the consumption of legumes, especially beans, and also of dairy products, mainly milk. Interestingly, our cohort showed an equol production ratio comparable to the observed 20–30% in Western countries rather than a higher production such as those observed in the Asian population [34,36]. Previous studies found that overweight/obese people were three times less likely to produce ODMA [48]. While the mean BMI of our participants was 29.7 (SD 8.09), which is in the category of overweight, our study revealed that the production of ODMA was lower (68%) than the 80–90% reported in the general population [43,45,46]. In our study, we also showed that most of the participants produced the non-flavonoid lignans enterodiol and enterolactone, which are phytoestrogens that have been studied as potential protective agents against breast cancer [59,60]. Both compounds are inhibitors of enzymes involved in steroid metabolism such as aromatase, 5 α-reductase, and 7β-hydroxysteroid dehydrogenase [59].

When we compared the phytoestrogen production and their metabolite correlations, equol had a statistically significant (*p* < 0.0001) correlation to ODMA (a daidzein metabolite), enterodiol (an enterolignan), and genistein (a soy isoflavone). Thus, in this healthy Puerto Rican cohort, there does not appear to be a daidzein and dihydrodaidzein correlation to equol production, which is a well-known daidzein metabolite. Our findings are similar to other studies and suggest that the lack of correlation could be linked to the consumption of other dietary components besides legumes or that not all legumes are a source of isoflavones [61]. Although we recruited our target sample size of 80 subjects, this presents a limitation because it is a relatively small sample size. This might have influenced the associations between each metabolite phenotype as well as the subsequent analysis for bacterial diversity. On the other hand, the strengths of the current study include the exclusion criteria to select a specific population. Our participants were not on antibiotic treatment and did not have an intestinal disease, which are factors that can affect gut diversity and subsequently the production of metabolites that may skew the classification of the different phenotypes.

Gut dysbiosis has been linked to several diseases including cancer [62]. In this study, we report that equol producers (among soy, bean, and high frequency dairy consumers) had reduced gut diversity and overall showed a lesser amount of beneficial gut bacteria (e.g., *Bacteriodes* spp. or *Acidominoccocus*), with higher dominance of *Prevotella*, and *Akkermansia* that are linked to people that have a dysbiotic gut. Accordingly, *Prevotella* and *Akkermansia* have been identified previously in other studies as equol-producing bacteria [63]. Cady et al. also showed that *Akkermansia* was found in greater abundance of subjects suffering from a chronic disease (i.e., multiple sclerosis), and they suggest that these gut microbes might promote pro-inflammatory effects that contribute to the severity of disease. Our study also identified other microbes in higher abundance among equol producers. These include *Lachnoclostridium*, *Enterobacteriaceae*, *Lachnospiraceae*, and *Coprobacter*. *Lachnoclostridium* was recently identified as an equol-producing bacteria in a study comparing the intestinal bacteria responsible for equol production in gut microbiota between equol producers and non-producers in Japan, in association with their daidzein intake [35]. *Enterobacteriaceace* was found among the equol producing bacteria in an in vitro study performed to find dietary conditions favoring endogenous equol biosynthesis using a pooled fecal homogenate from equol-producing women [64]. Bacteria from the *Lachnospiraceae* genus were found to be significantly increased in an equol-producing menopausal woman who was supplemented with long-term isoflavone treatment [65]. In this referenced study, there was a positive correlation of fecal *Lachnospiraceae* with the levels of equol excretion in urine. Moreover, *Prevotela*, *Dialister*, *Lachnospiraceae*, and *Coprobacter* are bacteria associated with the intake of dietary fiber [66]. However, in our current study, all of these bacteria genres were found in the fecal samples from healthy pre-menopausal equol-producing women and were associated with decreased gut health. 

*Bacteroides* proved to be mostly present in equol non-producers among soy and high frequency dairy consumers. *Bacteroides* have been associated with improving gut health, especially with reduced constipation, and protective of other diseases such as autism spectrum disorder [67]. Several species of *Bacteroides* are considered dominant beneficial bacteria because they metabolize polysaccharides and oligosaccharides and provide nutrition and vitamins to the host and other intestinal microbial residents [68]. We also detected *Bifidobacterial* dominance—a taxa that helps modulate gut microbiota and prevent inflammation [69], especially among those who rarely consume dairy or who do not consume soy.

## 5. Conclusions

In conclusion, our results suggest that healthy Puerto Rican women who are equol-producers are at higher risk for gut dysbiosis and their associated disease outcomes (e.g., cancer and other diseases) [70]. Most importantly, our data suggest a reduced gut diversity and higher dysbiosis for women that consume soy, are equol producers, and have a frequent dairy intake. This study serves as a starting point for future research to elucidate the role of equol on Puerto Ricans′ health, especially among breast cancer patients. Our results serve as fundamental knowledge in an understudied population and provide preliminary dietary guidelines to enhance gut health with the goal of preventing the development of highly prevalent and chronic diseases such as cancer.

## Figures and Tables

**Table 1:** Study participant anthropometrics, demographics, and lifestyle factors (*N* = 80).

Demographics and Anthropometrics	Mean ± SD
Age (years)	38.9 ± 7.8
Menarche (years)	12.3 ± 1.9
BMI	29.7 ± 8.1
**Education (highest degree earned)**	%
Elementary School	1.3
Intermediate School	16.3
High School	10
University	72.5
**Income (per year)**	
<$10,000	37.5
$20,000–$30,000	40
$30,000–$40,000	10
>$50,000	7.5
**Smoker**	
No	67.9
Yes	32.1
**Constipation**	
No	31.6
Yes	61.4
**Exercise**	
No	52.5
Yes	47.5
**Medical Insurance**	
No	5
Yes	95
**Type of Insurance**	
Government	31
Private	69
**Breast Cancer Family History**	
No	66.3
Yes	27.5
Do not know	6.3

**Table 2:** Percent consumption or non-consumption of top six foods in healthy Puerto Rican women (*N* = 80).

Food	Not Consume (%)	Consume (%)
Beans	13.8	86.2
Dairy	2.5	96.3
Fruit	13.8	86.2
Meat	3.8	96.2
Soy	58.8	41.2
Vegetables	15.0	85.0

**Table 3:** Correlation of phytoestrogen metabolites in the urine of healthy Puerto Rican women.

Metabolite	Metabolite Correlated to	r	*p* Value
ODMA	Enterodiol	0.287	0.0100
	Enterolactone	0.071	0.5320
	Daidzein	0.744	0.0001
	DHD	0.000	0.9990
	Equol	0.487	0.0001
	Genistein	0.885	0.0001
Daidzein	Enterodiol	0.054	0.6370
	Enterolactone	−0.019	0.8650
	DHD	0.284	0.0001
	Equol	0.151	0.1810
	Genistein	0.635	0.0001
Equol	Enterodiol	0.463	0.0001
	Enterolactone	0.145	0.2010
	DHD	−0.034	0.7630
	Genistein	0.605	0.0001
Genistein	Enterodiol	0.434	0.0100
	Enterolactone	0.102	0.3670
	DHD	0.074	0.5150
Enterodiol	Enterolactone	0.573	0.0001
	DHD	0.000	0.9990
Enterolactone	DHD	−0.058	0.6120

ODMA: O-desmethylangolensin, DHD: dihydrodaidzein.

## Data Availability

The 16S-rRNA reads were deposited in QIITA https://qiita.ucsd.edu/study/description/12663 and also in the EBI with accession number ERP129008.

## Supplementary Materials

The following are available online at https://www.mdpi.com/article/10.3390/ijerph18168254/s1, Figure S1: Equol non-producer bean consumers have a distinct community structure, Figure S2: Microbiota profiles among all participants according to their bean and soy consumption and equol production.
