# Supplementary figures and images for: Soy and Frequent Dairy Consumption with Subsequent Equol Production Reveals Decreased Gut Health in a Cohort of Healthy Puerto Rican Women
Source: Int J Environ Res Public Health. 2021 Aug 4;18(16):8254. doi: 10.3390/ijerph18168254 (PMC8391519; doi:10.3390/ijerph18168254)

**A**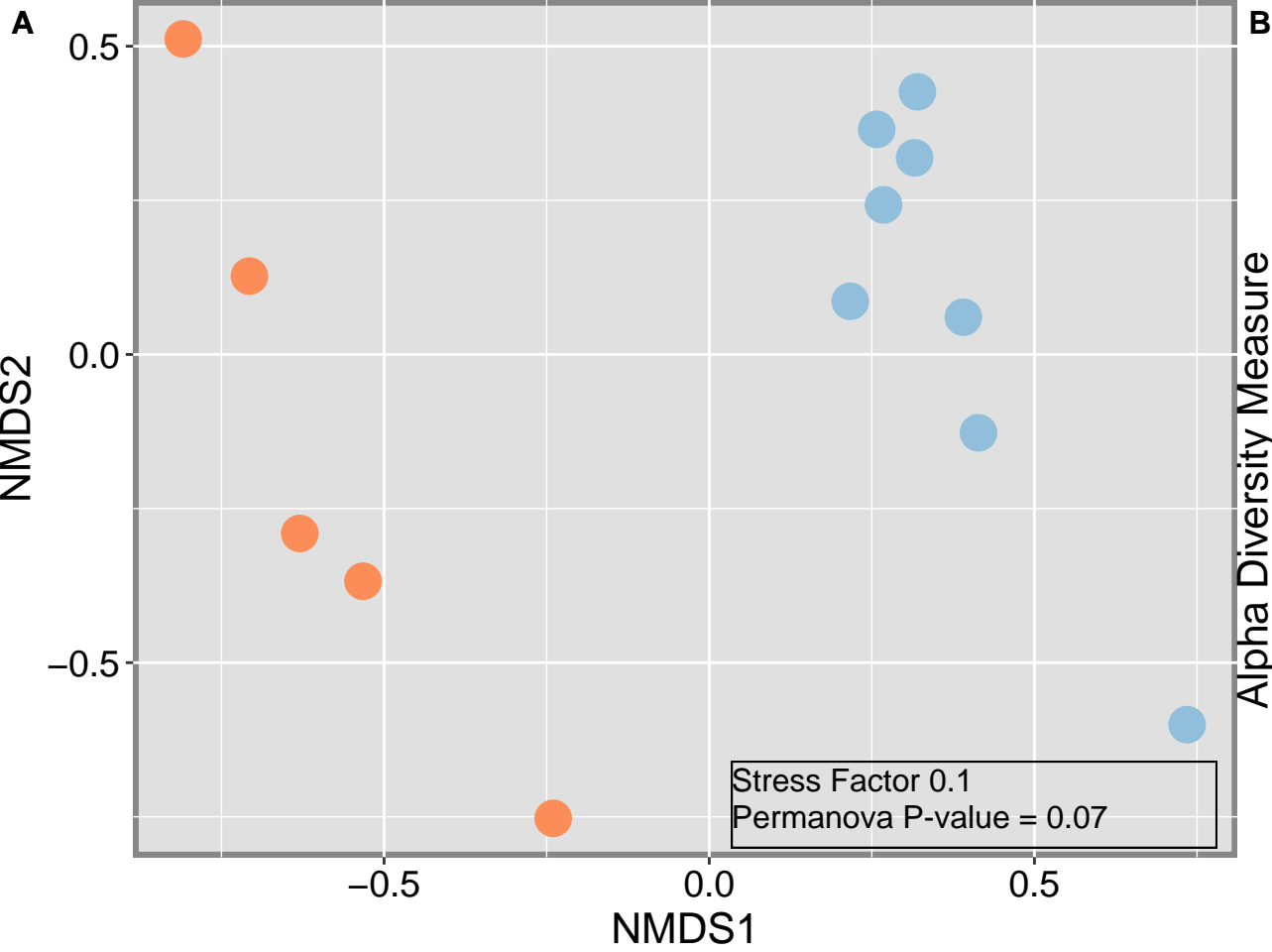**B**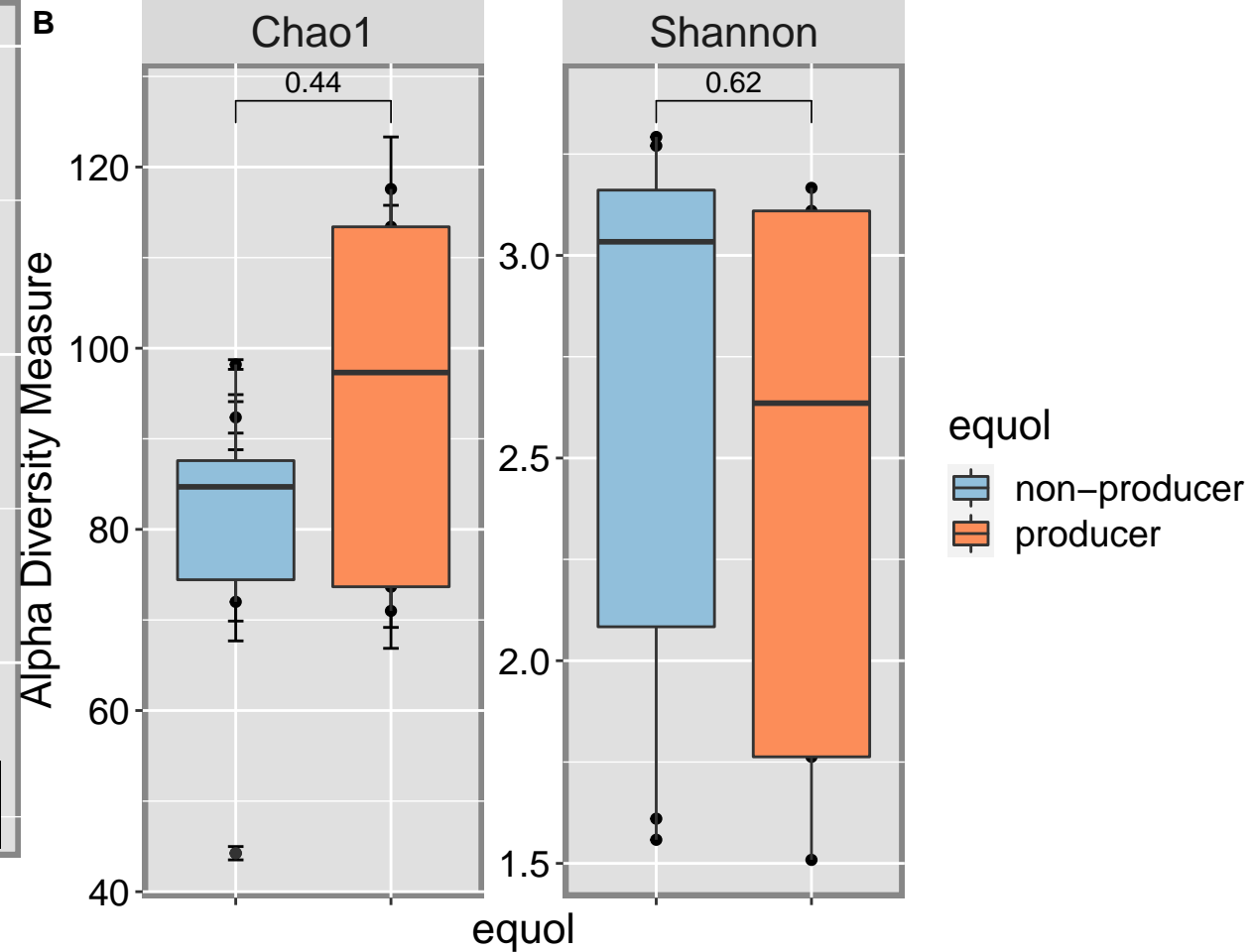

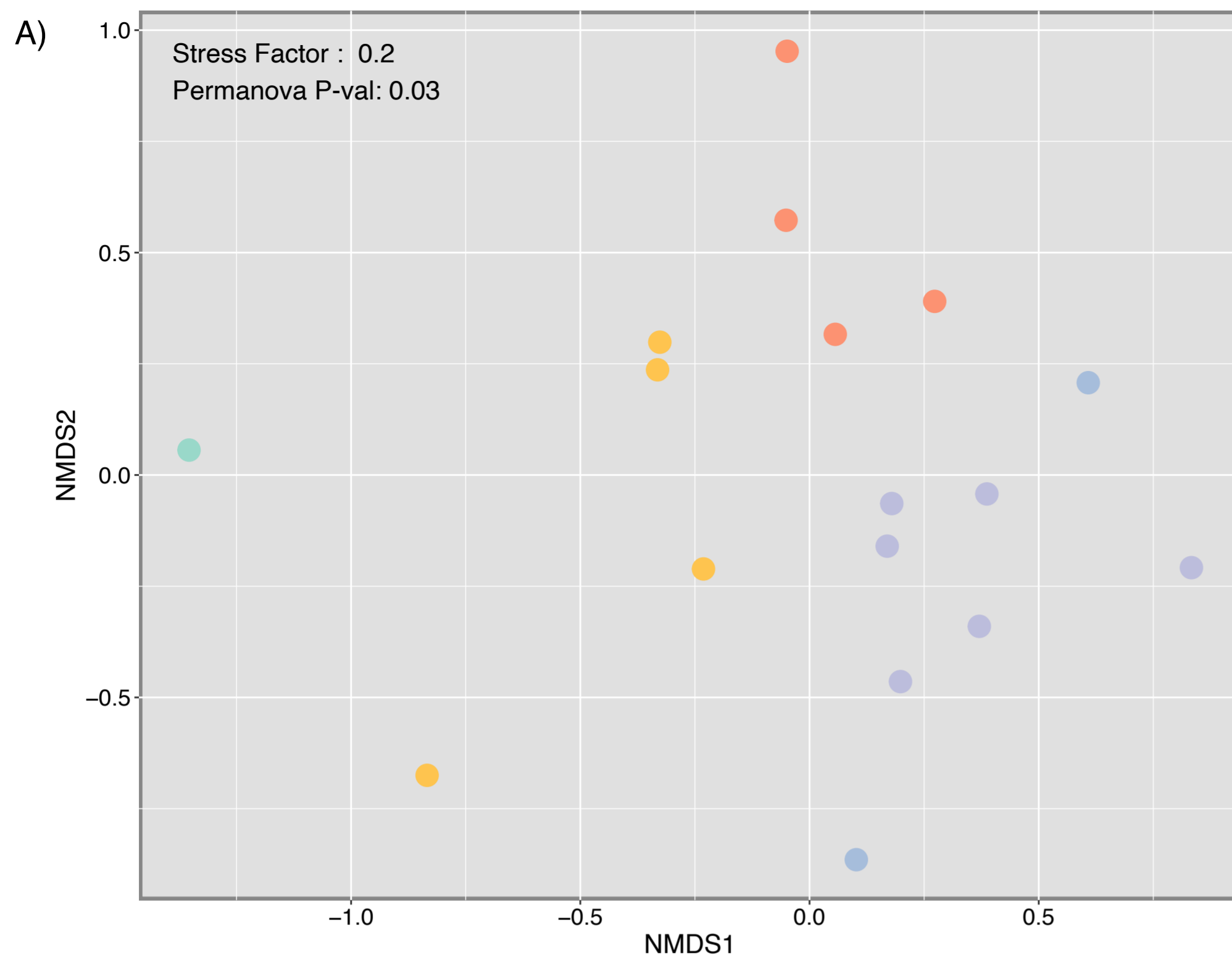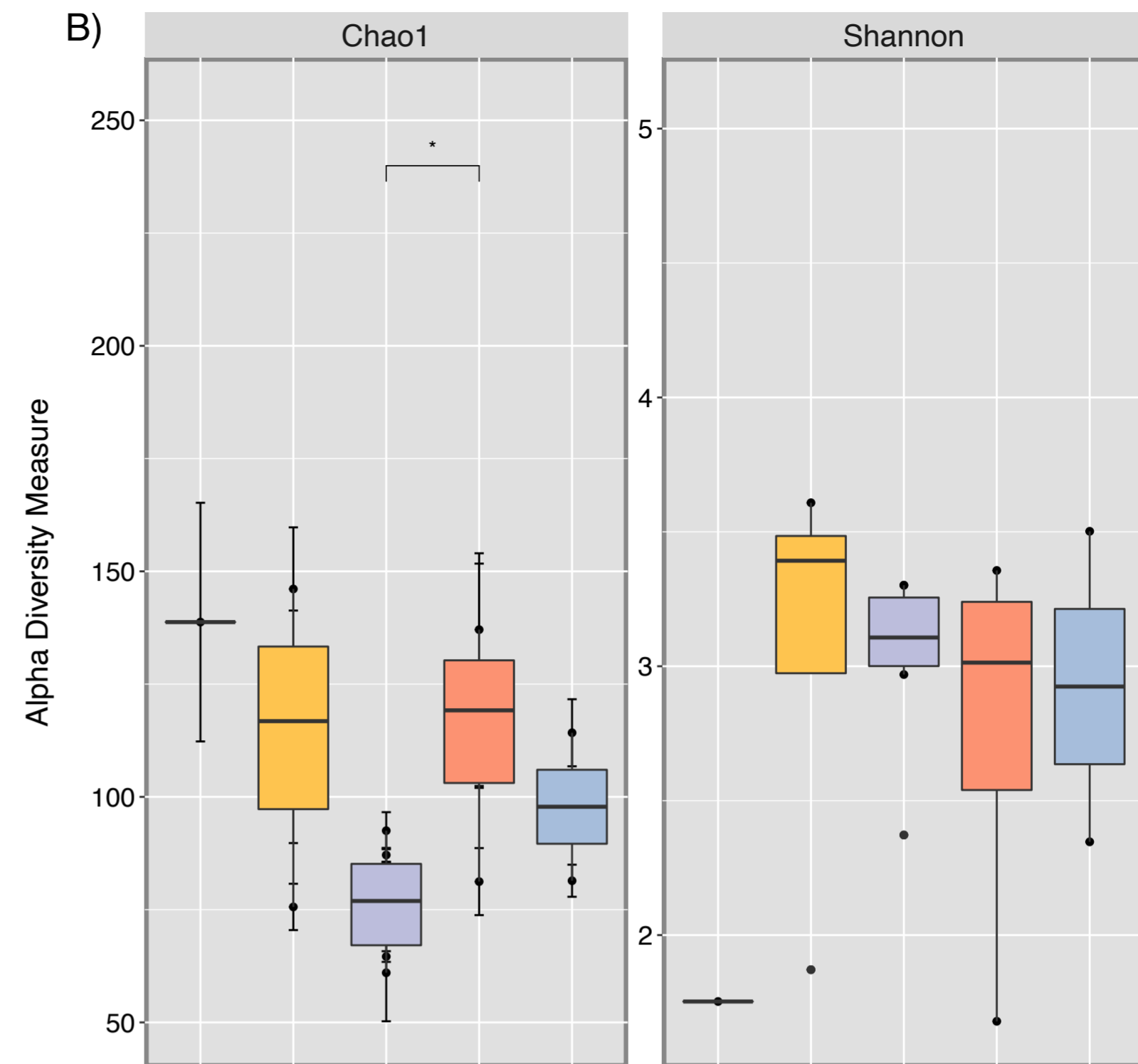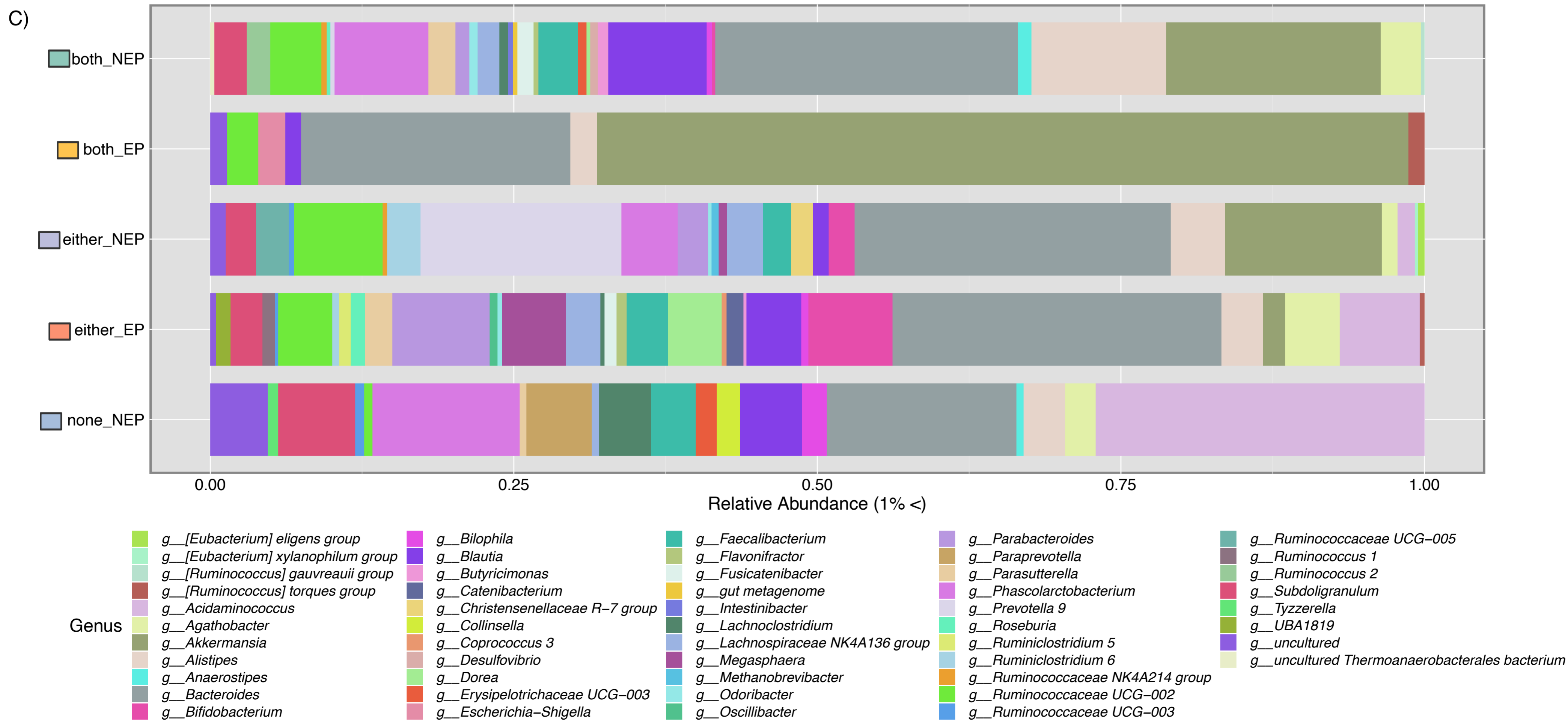

Supplement: Supplementary file 1 [file ijerph-18-08254-s001.zip › ijerph-1280061-supplementary.pdf]
